# Migrated fish bone into the neck: a case report

**DOI:** 10.1186/s13256-021-02968-2

**Published:** 2021-09-12

**Authors:** Vasanthika Sanjeewanie Thuduvage

**Affiliations:** Faculty of Medicine, General Sir John Kotelawala Defence University, Ratmalana, 10390 Sri Lanka

**Keywords:** Foreign body, Migrating, Complication, Case presentation, Lateral neck, Fish bone

## Abstract

**Background:**

Impaction of foreign body is a common condition presented to ear, nose, and throat department among Asian population. The commonest foreign body seen among this population has been documented as fish bone. Fish bone can migrate to lateral neck space or related organs around the neck and chest. By presenting this case report, we aim to emphasize the importance of taking proper history and make clinicians aware of the possibility of a fish bone migrating into different spaces. This will help to prevent diagnosis delay leading to complications due to migrated fish bone.

**Case presentation:**

A 50-year-old female Sinhalese patient presented to ear, nose, and throat department with right-sided neck pain for 2 days, who had a history of suspected fish bone impaction a few days ago that subsided without any investigations or treatments. She did not have any symptoms related to throat, and neck examination showed mild swelling and tenderness. Computer tomography revealed a migrated fish bone into the lateral neck close to carotid artery, and the fish bone was removed by neck exploration under general anesthesia without any complications.

**Conclusion:**

In conclusion, migrated fish bone should be suspected if patient is having persistent symptoms mainly in the neck without having difficulty swallowing and who gives a history of fish bone impaction and having negative laryngoscopic examination. Proper history taking is very important in the assessment of these patients to prevent misdiagnosis of the condition. Clinicians should aware that migrated fish bones are not uncommon and that early suspicion can prevent later diagnosis and complications.

## Background

Impaction of foreign body (FB) is a common condition among Asian population, and the commonest foreign body seen among this population has been documented as fish bone. This patient, who has had a history of fish bone impaction in the throat, presented later with neck pain and swelling and was later found to have migrated fish bone in the neck. If careful history had not been taken from the patient about history of fish bone impaction, this could have gone unnoticed by the clinicians. Neck pain and swelling could have been attributed to some other reason, such as infection, and fish bone could have gone unnoticed. By presenting this case report, we aim to emphasize the importance of taking proper history and make clinicians aware of the possibility of fish bone migrating into different spaces. There have been several occasions where fish bones migrated into neck, liver, and thyroid gland.

## Case presentation

**Fig. 1:**
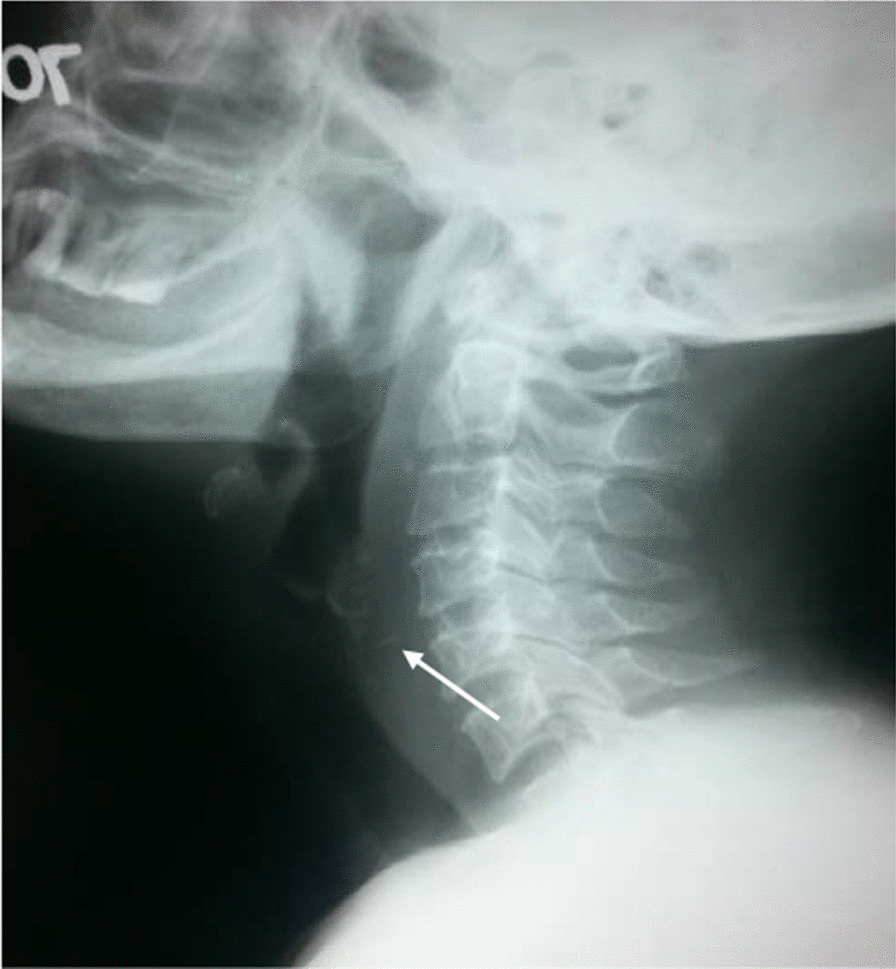
X-ray of the neck. Arrow pointing the fish bone in the X-Ray

**Fig. 2:**
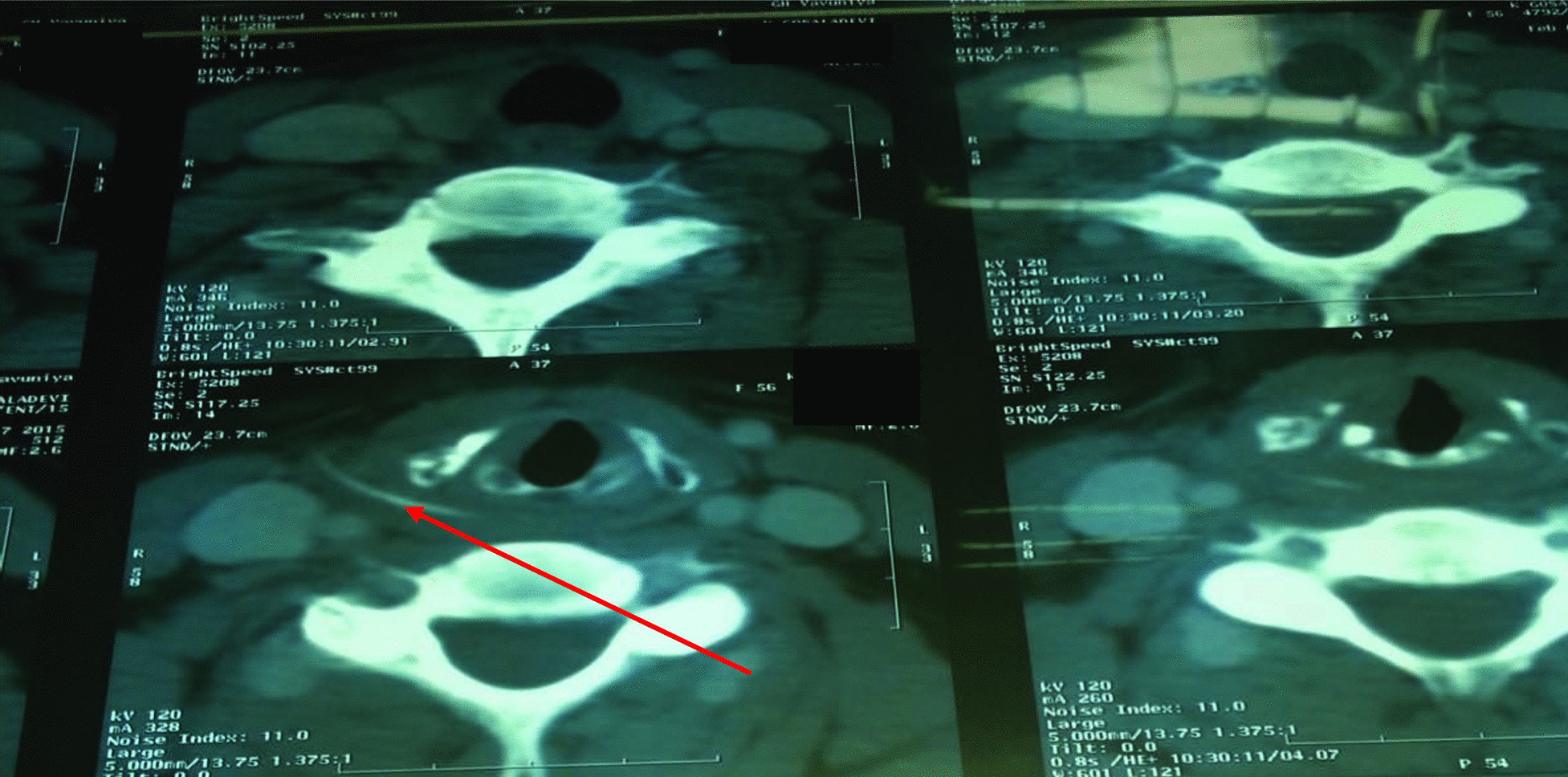
Computer Tomography image of neck showing the fish bone. Arrow pointing the fish bone in the Computer Tomography scan

A 50-year-old female Sinhalese patient presented to ear, nose, and throat department with right-sided neck pain for a period of 2 days. She gave a history of suspected fish bone in the throat a few days before where symptoms subsided with time without any investigations or treatments. She has had severe odynophagia initially and later resolved. She had no history of fever. Throat examination was normal including flexible laryngoscope examination of the throat. Neck examination showed mild swelling over the right lateral neck, and tenderness was elicited over the right-sided lateral neck. X-ray lateral neck soft-tissue view (Fig. 1) examination showed a suspicions opacity over the esophageal shadow area. Ultrasound scan results indicated likely parapharyngeal infection of right side of neck. As her swallowing was normal even though the X-ray showed a suspicious opacity, computed tomography (CT) scan was arranged. CT shows (Fig. 2) a sharp fish bone migrated to the right side of the lateral neck very close to the right carotid artery.

**Fig. 3:**
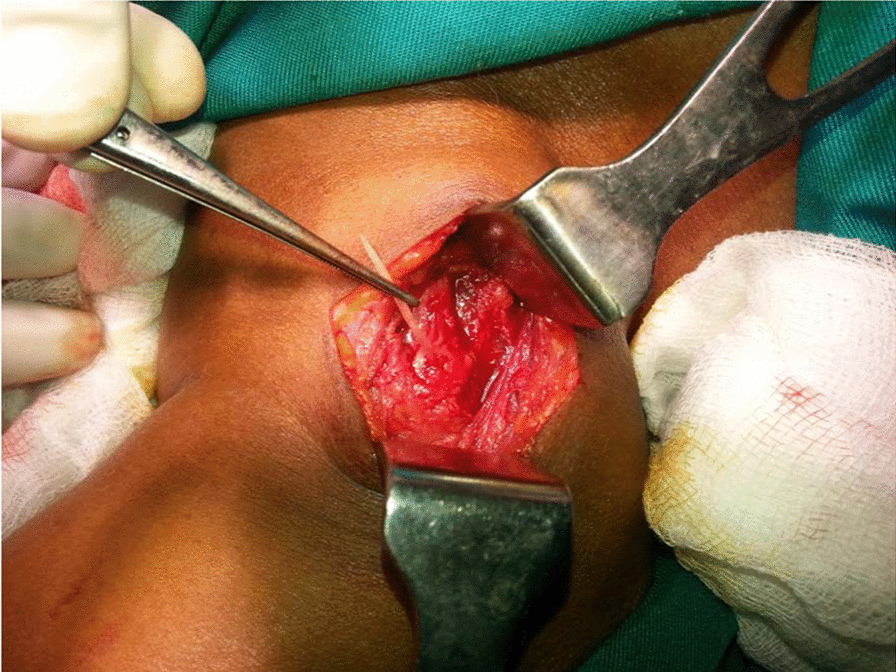
Neck exploration

**Fig. 4:**
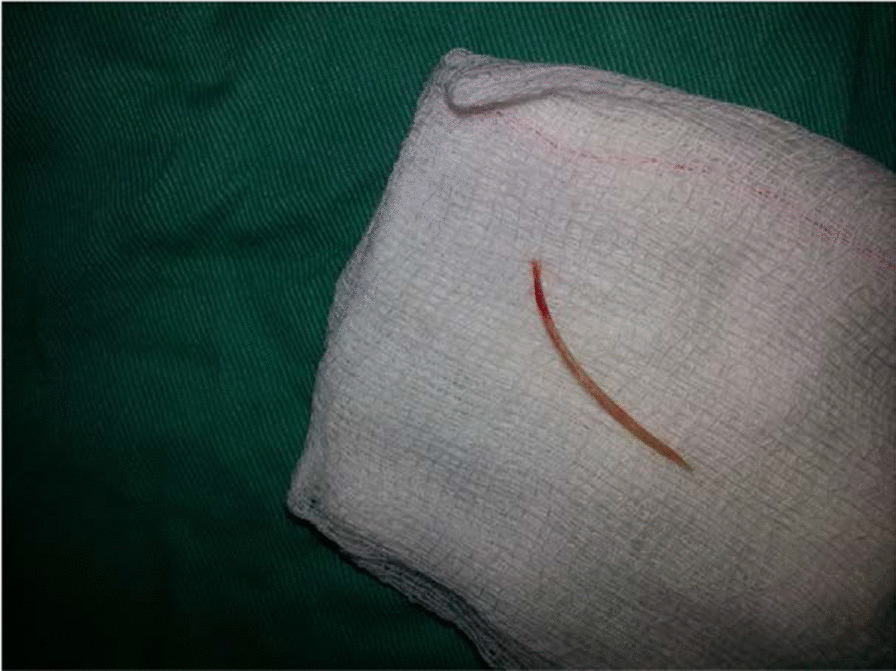
Fish bone after removal

After getting informed written consent, neck exploration was planned under general anesthesia. The patient was positioned supine with a neck extension and rotated to left side. Incision was made over the right lateral neck at the site of maximum swelling with the guidance of CT findings. Careful exploration of the area was done after lateralizing the sternomastoid muscle. Fish bone was found under the sternomastoid muscle (Fig. 3) lateral to the esophagus. Fish bone was removed (Fig. 4), and hemostasis was achieved. Some inflammation around the impaction site was noted, but no pus collection was noted. A suction drain was inserted, and wound was primarily closed. The patient was given intravenous antibiotics. The patient was followed up in the clinic for 3 months after discharge. The patient did not have any immediate postoperative complications or long-term complications after the procedure.

## Discussion and conclusion

Foreign body impaction in the throat is a very common condition and a common presenting complaint at accident and emergency department. Fish bone is the commonest FB that clinicians encountered in FB impacted patients in ear, nose, and throat practice especially in Asian population [3]. Clinicians experience the same trend in Sri Lanka. Other FB that people get impacted include chicken bone, sea food parts such as prawn parts, coins in pediatric age group, and metallic covers of medicine tablets. In some instances, toy batteries have been the culprit in pediatric population.

The commonest areas of fish bone impaction in throat are tonsils, tongue base, vallecular area, pyriform fossa, and cricopharyngeal area. Fish bones impacted on tonsils and tongue base can be removed at outpatient department without any adverse side effects or complications [3]. Sometimes flexible laryngoscope helps in removing fish bone using the foreign body removal forces. It has been documented that FB impacted at the cricopharynx or at any narrow point of esophagus accounts for 5% of cases [5].

In most instances, patients with impacted foreign bodies presented within 24 hours [4]. Unwanted serious complications have been documented due to delay in presentation [4]. Symptoms include painful swallowing (odynophagia) and severe sharp pain on swallowing [1]. Proper history taking and examination to see the fish bone will reveal important signs to manage the patient. Laryngoscopic examination and rigid esophagoscopic examination are important procedures. If they are negative and patient continues to have persistent symptoms, further imaging studies will help to rule out migrated fish bones. The FB can migrate into surrounding structures extraluminally and cause symptoms with negative endoscopic findings. There have been incidences where migrated fish bone appeared from patient’s shoulder area and was removed under local anesthesia [9].

Diagnosis depends on proper history taking and findings of throat examination. Flexible and rigid endoscopy procedures are useful procedures to find the fish bone and plan for removal. X-ray neck soft-tissue view (lateral and anteroposterior view) will help to locate a radio opaque FB including fish bone and meat bone. Sensitivity and specificity of X-ray has been reported as 39% and 72%, respectively, in relation to detection of fish bone [6].

In some instances, fish bone will penetrate the mucosal wall, migrate to the surrounding structures, and be found in the neck or chest. If a patient has persisting symptoms and a positive feature on neck radiography with negative laryngoscopic examination and rigid endoscopy, clinician should have a high index of suspicion about the possibility of migrated fish bone. Migrated fish bone can be complicated with neck abscess vascular complications and retention in thyroid gland [7], and pseudotumor of liver has been reported in rare occasions when the fish bone has migrated to the gastrointestinal tract [8]. Very rarely, fish bones were found in the thyroid gland [2]. Ultrasound scan of neck may be beneficial in some instances with neck abscess as a complication. CT scan has a role in situations where complications are seen related to the fish bone impaction or having negative investigation modalities with positive symptoms. Details related to the shape of the fish bone, site of the fish bone and relations of the fish bone to the surrounding structures can be obtained from a CT scan. In a case of neck abscess, it can be also use to locate the site of the abscess and measure the size of the abscess. CT scan will play an important role in diagnosis of these patients, and it will help the surgeon during neck exploration. Considering FB retention in the thyroid gland, ultrasonography neck is superior to CT scan in terms of differentiating the calcification from the FB. Besides, ultrasonography was able to show the shape and orientation of the fish bone after several directional changes of the probe [7]. Neck ultrasonography findings of our patient were not conclusive.

A common reason for migration of fish bone is related to shape of the bone, sharpness and direction of the bone, or orientation of the bone. Migration of FB will be influenced by several factors, such as shape of the FB and orientation of the FB [4]. In our case, the fish bone was a quite sharp and long, having a pointed end. Those features should have facilitated the migration of the FB into the neck space

Management of these patients depends on where the fish bone is located and associated complications such as abscess. Surgical exploration is the best modality of management in most situations, and careful identification of anatomical landmarks is important to prevent vascular damage during the procedure.

Sri Lankans usually try several remedial practices to dislodge a fish bone once they get an impacted fish bone. Those include swallowing big boluses of rice and swallowing big portion of banana. This type of practice will facilitate migration of the fish bone into the extraluminal space in the neck.

Late identification of migrated fish bone will lead to life-threatening complications, and outcome will not be acceptable. They will present with severe retropharyngeal abscess with airway compromise or neck abscess leading to mediastinitis. Neck inflammation can be worsened by the presence of sepsis or use of radiation, which may worsen the symptoms of fish bone impaction.

All these cases need to be investigated with CT scan, and early treatment is needed.

In conclusion, migrated fish bone should be suspected if patient is having persistent symptoms mainly in the neck without having difficulty swallowing who gives a history of fish bone impaction and having negative laryngoscopic examination. Proper history taking is very important in the assessment of these patients to prevent misdiagnosis of the condition. Plain X-ray neck lateral view may be either negative or positive depending on the radiolucent or radiopaque nature of the fish bone. CT scan is the best diagnostic tool, helping to determine the exact nature and position of the fish bone. Neck ultrasonography will be a useful aid to be used with the CT scan. Early diagnosis and proper planning of surgical exploration without delay will facilitate the prevention of life-threatening consequences.

## Data Availability

Data sharing is not applicable to this article because no datasets were generated or analyzed during the current study.

## Abbreviations

FB: Foreign body
CT: Computer tomography
